# *catena*-Poly[[[(2,2′-bipyridine-κ^2^ *N*,*N*′)manganese(II)]-μ-(2,5-dichloro-3,6-dioxocyclo­hexa-1,4-diene-1,4-diolato)-κ^4^ *O* ^1^,*O* ^6^:*O* ^3^,*O* ^4^] ethanol disolvate]

**DOI:** 10.1107/S1600536813001438

**Published:** 2013-01-19

**Authors:** Yuji Nishimura, Akiko Himegi, Akira Fuyuhiro, Shinya Hayami, Satoshi Kawata

**Affiliations:** a Department of Chemistry, Faculty of Science, Fukuoka University, Nanakuma, Jonan-ku, Fukuoka 814-0180, Japan; b Department of Chemistry, Graduate School of Science, Osaka University, Toyonaka, Osaka 560-0043, Japan; c Department of Chemistry, Graduate School of Science and Technology, Kumamoto University, Kurokami, Kumamoto 860-8555, Japan

## Abstract

The asymmetric unit of the title coordination polymer, {[Mn(C_6_Cl_2_O_4_)(C_10_H_8_N_2_)]·2C_2_H_5_OH}_*n*_, consists of one Mn^II^ ion, one 2,2′-bipyridine (bpy) ligand, one chloranilate (CA^2−^) ligand and two ethanol solvent mol­ecules. The Mn^II^ ion is octa­hedrally coordinated by two N atoms of one bpy ligand and four O atoms of two chloranilate ions. The chloranilate ion serves as a bridging ligand between the Mn^II^ ions, leading to an infinite zigzag chain along [101]. π–π stacking inter­actions [centroid–centroid distance = 4.098 (2) Å] is observed between the pyridine rings of adjacent chains. The ethanol mol­ecules act as accepters as well as donors for O—H⋯O hydrogen bonds, and form a hydrogen-bonded chain along the *a* axis. The H atoms of the hy­droxy groups of the two independent ethanol mol­ecules are each disordered over two sites with equal occupancies.

## Related literature

For related structures, see: Nagayoshi *et al.* (2003 ▶); Decurtins *et al.* (1996 ▶); Deguenon *et al.* (1990 ▶); Kabir *et al.* (2001 ▶); Zheng *et al.* (1996 ▶).
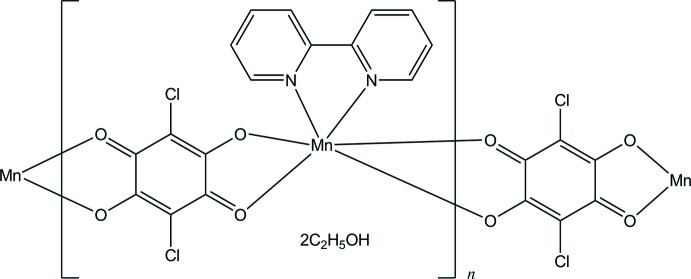


## Experimental

### 

#### Crystal data

[Mn(C_6_Cl_2_O_4_)(C_10_H_8_N_2_)]·2C_2_H_6_O
*M* *_r_* = 510.22
Monoclinic, 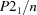

*a* = 8.3130 (15) Å
*b* = 20.866 (4) Å
*c* = 12.513 (2) Å
β = 97.665 (2)°
*V* = 2151.2 (7) Å^3^
*Z* = 4
Mo *K*α radiation
μ = 0.90 mm^−1^
*T* = 100 K
0.40 × 0.10 × 0.05 mm

#### Data collection

Rigaku Saturn724 diffractometer
Absorption correction: multi-scan (*REQAB*; Rigaku, 1998 ▶) *T*_min_ = 0.897, *T*_max_ = 0.956
24503 measured reflections
4903 independent reflections
4526 reflections with *I* > 2σ(*I*)
*R*_int_ = 0.028

#### Refinement

*R*[*F* ^2^ > 2σ(*F* ^2^)] = 0.037
*wR*(*F* ^2^) = 0.089
*S* = 1.10
4903 reflections
284 parameters
H-atom parameters constrained
Δρ_max_ = 0.99 e Å^−3^
Δρ_min_ = −0.57 e Å^−3^

### 

Data collection: *CrystalClear* (Rigaku, 2008 ▶); cell refinement: *CrystalClear*; data reduction: *CrystalClear*; program(s) used to solve structure: *Il Milione* (Burla *et al.*, 2007 ▶); program(s) used to refine structure: *SHELXL97* (Sheldrick, 2008 ▶); molecular graphics: *CrystalStructure* (Rigaku, 2010 ▶); software used to prepare material for publication: *CrystalStructure* (Rigaku, 2010 ▶).

## Supplementary Material

Crystal structure: contains datablock(s) I, global. DOI: 10.1107/S1600536813001438/is5235sup1.cif

Structure factors: contains datablock(s) I. DOI: 10.1107/S1600536813001438/is5235Isup2.hkl

Additional supplementary materials:  crystallographic information; 3D view; checkCIF report

## Figures and Tables

**Table 1:** Selected bond lengths (Å)

Mn1—O1	2.1796 (14)
Mn1—O2	2.1546 (14)
Mn1—O3^i^	2.1511 (14)
Mn1—O4^i^	2.1782 (14)
Mn1—N1	2.2473 (16)
Mn1—N2	2.2398 (16)

Symmetry code: (i) 
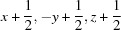
.

**Table 2:** Hydrogen-bond geometry (Å, °)

*D*—H⋯*A*	*D*—H	H⋯*A*	*D*⋯*A*	*D*—H⋯*A*
O5—H1⋯O6	0.84	1.91	2.716 (4)	160
O5—H4⋯O5^ii^	0.84	2.00	2.715 (4)	142
O6—H2⋯O6^iii^	0.84	1.83	2.661 (3)	170
O6—H3⋯O5	0.84	1.90	2.716 (4)	162

Symmetry codes: (ii) 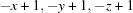; (iii) 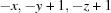.

**Fig. 1.**
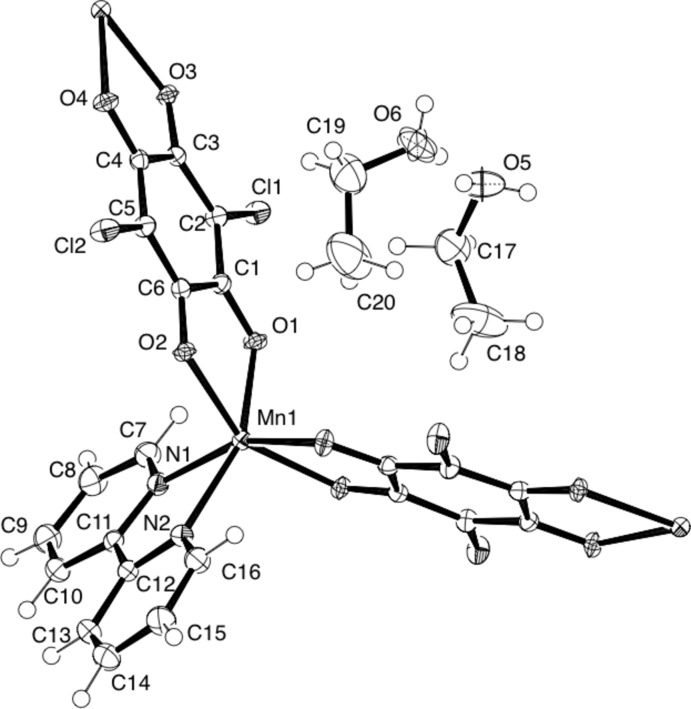
An ORTEP drawing for the title compound, showing 50% probability displacement ellipsoids.

**Fig. 2.**
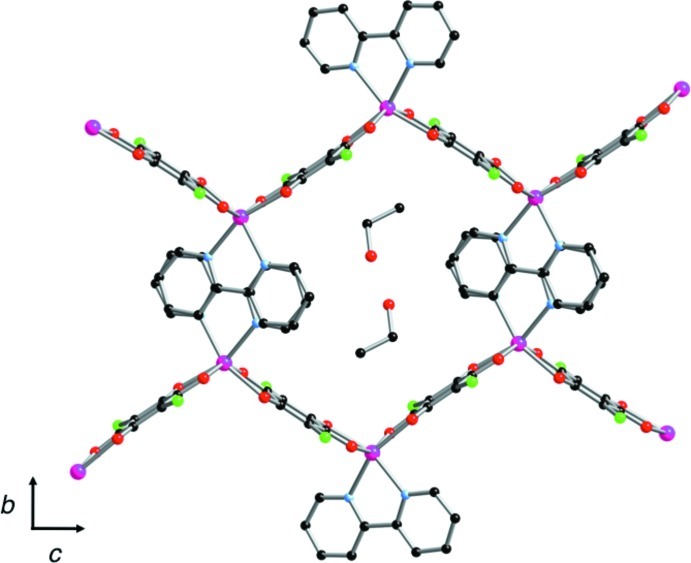
A fragment of one-dimensional channel structure of the title compound along the a axis. H atoms have been omitted for clarity.

**Fig. 3.**
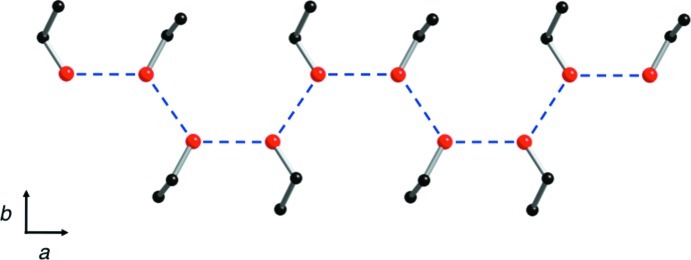
A partial packing view of the title compound, showing the ethanol solvent molecules forming a one-dimensional chain along the a axis through hydrogen bonds. The hydrogen bonds are shown as dashed lines. H atoms have been omitted for clarity.

## supplementary crystallographic information

### Comment

In this paper manganese assembled structures of chloranilic acid (H_2_CA = 2,5-dichloro-3,6-dihydroxy-1,4-benzoquinone) are rationally designed by using bpy. Chloranilic acid can coordinate to metal ions in both the bidentate and the bisbidentate fashions (Nagayoshi *et al.*, 2003). The dianion of chloranilic acid consists of two allyl systems connected by C—C single bonds, with four oxygen atoms partially negatively charged. This potentiality allows for the coordination of transition-metal ions through CA^2-^ bridges and permits the probable propagation of magnetic super-exchange interactions between the paramagnetic centers. These kind of complexes using manganese two ions and H_2_CA were reported previously (Kabir *et al.*, 2001). We report here, {[Mn(C_10_H_8_N_2_)(C_6_Cl_2_O_4_)].(C_2_H_6_O)_2_}*_n_* (1), which consists of the Mn(II) one-dimensional chain complex and two ethanol solvent molecules. The manganese(II) ion has a distorted octahedral environment, caused by fairly small bite angles of N—Mn—N [73.06 (7)°] and O—Mn—O [74.62 (5), 74.60 (5)°]. The latter compares with that in [Mn(bpy)CA]_n_ (2) (Zheng, *et al.*, 1996) [73.67 (7)°] but is smaller than that of O—Cu—O [77.31 (4)°] in [Cu(DCMB)(CA)]_n_ (DCMB = 3,3'-dicarbomethoxy-2,2'-bipyridine) (Decurtins *et al.*, 1996). The Mn—N distances [2.2472 (18) and 2.2397 (18) Å] agree well with those in [Mn(bpy)(C_2_O_4_)]_n_ (Deguenon *et al.*, 1990), (2.241, 2.258 Å) and the average Mn—O length (2.166 Å) is compatible with that in 2 (2.180 Å). Overall, the determined Mn—N and Mn—O bond lengths are in agreement with dipositive charged manganese ions as coordination centres. The CA^2-^ bridges Mn(II) ions, which leads to infinite chains exhibiting a zig-zag pattern with bipyridine ligands stacking between the chains. The nearest C–C distance of the stacked bipyridine ligands is 3.607 (3) Å. This stacking interaction makes two-dimensional packing structure. This Mn···Mn [8.131 (1) Å] separation is a little smaller than the Mn···Mn [8.170 Å] separation in the chain of 2. The chain complex was assembled in the *bc* plane to form a one-dimensional channel along the *a* axis. The crystal structures of 1, 2 and [Mn(CA)(terpy)]_n_ (terpy = 2,2:6,2-terpyridine) are similar. However, only compound 1 contains two ethanol solvents as solvent molecules. Interstitial solvents are introduced to the channel constructed by the assembling of one-dimensional chains to make a clathrate. Two ethanol solvent molecules are connected through hydrogen bonding, and form a one-dimensional chain along the *a* axis. As a result, voids of compound 1 is expanded by introduction of solvents into the clathrate.

### Experimental

A mixture of MnCl_2_.4H_2_O (1 ml, 5 mmol*L*^-1^) in aqueous solution and 2,2'-bipyridine (1 ml, 5 mmol*L*^-1^) in ethanol solution was transferred to a glass tube, and then an ethanol solution (10 ml) of H_2_CA (2 ml, 5 mmol*L*^-1^) was poured into the tube without mixing the two solutions. Dark violet crystals began to form at ambient temperature in a one week. One of these crystals was used for X-ray crystallography.

### Refinement

The C-bound H atoms in the bpy and the methyl group of the ethanol molecule were placed at calculated positions with C—H = 0.95 and 0.98 Å, respectively, and were treated as riding on their parent atoms with *U*_iso_(H) set to 1.2*U*_eq_(C). Both of the hydrogen atoms on the hydroxy groups of the ethanol solvent molecules are disordered over two sites, each with an occupancy of 0.5 and were treated as riding on their parent oxygen atoms, with O—H = 0.84 Å and with *U*_iso_(H) set to 1.5*U*_eq_(O).

### Crystal data

**Table:**

[Mn(C_6_Cl_2_O_4_)(C_10_H_8_N_2_)]·2C_2_H_6_O	*F*(000) = 1044
*M**_r_* = 510.22	*D*_x_ = 1.575 Mg m^−^^3^
Monoclinic, *P*2_1_/*n*	Mo *K*α radiation, λ = 0.71075 Å
Hall symbol: -P 2yn	Cell parameters from 6299 reflections
*a* = 8.3130 (15) Å	θ = 3.2–27.5°
*b* = 20.866 (4) Å	µ = 0.90 mm^−^^1^
*c* = 12.513 (2) Å	*T* = 100 K
β = 97.665 (2)°	Platelet, violet
*V* = 2151.2 (7) Å^3^	0.40 × 0.10 × 0.05 mm
*Z* = 4	

### Data collection

**Table:**

Rigaku Saturn724 diffractometer	4903 independent reflections
Radiation source: fine-focus sealed tube	4526 reflections with *I* > 2σ(*I*)
Graphite monochromator	*R*_int_ = 0.028
Detector resolution: 7.111 pixels mm^-1^	θ_max_ = 27.5°, θ_min_ = 3.2°
ω scans	*h* = −10→10
Absorption correction: multi-scan (REQAB; Rigaku, 1998)	*k* = −27→26
*T*_min_ = 0.897, *T*_max_ = 0.956	*l* = −16→16
24503 measured reflections	

### Refinement

**Table:**

Refinement on *F*^2^	Primary atom site location: structure-invariant direct methods
Least-squares matrix: full	Secondary atom site location: difference Fourier map
*R*[*F*^2^ > 2σ(*F*^2^)] = 0.037	Hydrogen site location: inferred from neighbouring sites
*wR*(*F*^2^) = 0.089	H-atom parameters constrained
*S* = 1.10	*w* = 1/[σ^2^(*F*_o_^2^) + (0.0369*P*)^2^ + 2.5077*P*] where *P* = (*F*_o_^2^ + 2*F*_c_^2^)/3
4903 reflections	(Δ/σ)_max_ = 0.002
284 parameters	Δρ_max_ = 0.99 e Å^−^^3^
0 restraints	Δρ_min_ = −0.57 e Å^−^^3^

### Special details

**Table:**

Geometry. All e.s.d.'s (except the e.s.d. in the dihedral angle between two l.s. planes) are estimated using the full covariance matrix. The cell e.s.d.'s are taken into account individually in the estimation of e.s.d.'s in distances, angles and torsion angles; correlations between e.s.d.'s in cell parameters are only used when they are defined by crystal symmetry. An approximate (isotropic) treatment of cell e.s.d.'s is used for estimating e.s.d.'s involving l.s. planes.
Refinement. Refinement of *F*^2^ against ALL reflections. The weighted *R*-factor *wR* and goodness of fit *S* are based on *F*^2^, conventional *R*-factors *R* are based on *F*, with *F* set to zero for negative *F*^2^. The threshold expression of *F*^2^ > σ(*F*^2^) is used only for calculating *R*-factors(gt) *etc*. and is not relevant to the choice of reflections for refinement. *R*-factors based on *F*^2^ are statistically about twice as large as those based on *F*, and *R*- factors based on ALL data will be even larger.

### Fractional atomic coordinates and isotropic or equivalent isotropic displacement parameters (Å^2^)

**Table:**

	*x*	*y*	*z*	*U*_iso_*/*U*_eq_	Occ. (<1)
Mn1	0.26466 (3)	0.152282 (13)	0.52153 (2)	0.01343 (8)	
Cl1	0.34709 (5)	0.29498 (2)	0.19487 (4)	0.01890 (11)	
Cl2	−0.31626 (5)	0.22151 (2)	0.36711 (4)	0.02093 (11)	
O1	0.29779 (16)	0.21863 (6)	0.39171 (11)	0.0173 (3)	
O2	0.02350 (16)	0.18152 (6)	0.45478 (10)	0.0160 (3)	
O3	0.00620 (16)	0.32640 (6)	0.09766 (10)	0.0157 (3)	
O4	−0.26694 (16)	0.29681 (7)	0.16927 (11)	0.0180 (3)	
O5	0.4059 (3)	0.44845 (11)	0.46828 (18)	0.0517 (5)	
H1	0.3160	0.4420	0.4900	0.078*	0.50
H4	0.4601	0.4714	0.5151	0.078*	0.50
O6	0.0886 (3)	0.44697 (10)	0.49882 (18)	0.0513 (5)	
H2	0.0270	0.4791	0.4920	0.077*	0.50
H3	0.1820	0.4395	0.4833	0.077*	0.50
N1	0.32635 (19)	0.07145 (8)	0.41547 (13)	0.0169 (3)	
N2	0.19315 (19)	0.06124 (8)	0.59751 (13)	0.0169 (3)	
C1	0.1713 (2)	0.23827 (9)	0.33539 (14)	0.0141 (3)	
C2	0.1669 (2)	0.27578 (9)	0.24228 (15)	0.0147 (3)	
C3	0.0202 (2)	0.29465 (8)	0.18379 (14)	0.0132 (3)	
C4	−0.1402 (2)	0.27663 (8)	0.22528 (15)	0.0139 (3)	
C5	−0.1360 (2)	0.23993 (9)	0.31882 (15)	0.0153 (3)	
C6	0.0100 (2)	0.21788 (8)	0.37393 (14)	0.0142 (3)	
C7	0.3914 (3)	0.07952 (10)	0.32347 (16)	0.0221 (4)	
H7	0.4180	0.1217	0.3031	0.026*	
C8	0.4214 (3)	0.02880 (11)	0.25711 (17)	0.0264 (4)	
H8	0.4677	0.0361	0.1927	0.032*	
C9	0.3826 (3)	−0.03242 (11)	0.28672 (18)	0.0275 (5)	
H9	0.4012	−0.0680	0.2425	0.033*	
C10	0.3160 (3)	−0.04169 (10)	0.38176 (17)	0.0233 (4)	
H10	0.2891	−0.0835	0.4036	0.028*	
C11	0.2895 (2)	0.01144 (9)	0.44437 (15)	0.0166 (4)	
C12	0.2186 (2)	0.00567 (9)	0.54702 (15)	0.0167 (4)	
C13	0.1817 (3)	−0.05325 (10)	0.58966 (17)	0.0228 (4)	
H13	0.1999	−0.0919	0.5530	0.027*	
C14	0.1180 (3)	−0.05466 (11)	0.68661 (18)	0.0279 (5)	
H14	0.0931	−0.0944	0.7175	0.033*	
C15	0.0909 (3)	0.00214 (11)	0.73776 (18)	0.0271 (5)	
H15	0.0468	0.0022	0.8040	0.033*	
C16	0.1296 (3)	0.05905 (10)	0.69034 (16)	0.0222 (4)	
H16	0.1102	0.0982	0.7251	0.027*	
C17	0.4877 (4)	0.38834 (13)	0.4599 (2)	0.0414 (6)	
H17A	0.5837	0.3952	0.4222	0.050*	
H17B	0.4138	0.3584	0.4158	0.050*	
C18	0.5409 (5)	0.35873 (16)	0.5669 (3)	0.0669 (11)	
H18A	0.5981	0.3185	0.5571	0.080*	
H18B	0.4458	0.3499	0.6032	0.080*	
H18C	0.6138	0.3882	0.6110	0.080*	
C19	−0.0048 (5)	0.39086 (16)	0.4859 (3)	0.0585 (8)	
H19A	−0.0062	0.3750	0.4113	0.070*	
H19B	−0.1180	0.4012	0.4963	0.070*	
C20	0.0558 (6)	0.33965 (17)	0.5616 (3)	0.0766 (12)	
H20A	−0.0281	0.3067	0.5625	0.092*	
H20B	0.0828	0.3577	0.6341	0.092*	
H20C	0.1531	0.3204	0.5385	0.092*	

### Atomic displacement parameters (Å^2^)

**Table:**

	*U*^11^	*U*^22^	*U*^33^	*U*^12^	*U*^13^	*U*^23^
Mn1	0.01400 (15)	0.01304 (14)	0.01278 (14)	0.00029 (10)	0.00001 (10)	−0.00026 (10)
Cl1	0.0120 (2)	0.0238 (2)	0.0214 (2)	−0.00032 (16)	0.00420 (16)	0.00607 (17)
Cl2	0.0124 (2)	0.0278 (2)	0.0233 (2)	0.00060 (17)	0.00476 (17)	0.00960 (18)
O1	0.0127 (6)	0.0198 (7)	0.0188 (6)	0.0002 (5)	0.0003 (5)	0.0053 (5)
O2	0.0151 (6)	0.0169 (6)	0.0157 (6)	0.0001 (5)	0.0015 (5)	0.0043 (5)
O3	0.0147 (6)	0.0172 (6)	0.0150 (6)	−0.0001 (5)	0.0012 (5)	0.0029 (5)
O4	0.0134 (6)	0.0224 (7)	0.0181 (6)	0.0009 (5)	0.0011 (5)	0.0060 (5)
O5	0.0511 (12)	0.0525 (12)	0.0534 (12)	0.0145 (10)	0.0140 (10)	0.0084 (10)
O6	0.0492 (12)	0.0384 (10)	0.0683 (14)	−0.0091 (9)	0.0155 (11)	0.0082 (10)
N1	0.0168 (8)	0.0171 (8)	0.0164 (7)	0.0001 (6)	0.0008 (6)	−0.0016 (6)
N2	0.0171 (8)	0.0160 (7)	0.0174 (7)	0.0005 (6)	0.0013 (6)	0.0004 (6)
C1	0.0127 (8)	0.0139 (8)	0.0153 (8)	0.0002 (6)	0.0009 (6)	−0.0009 (7)
C2	0.0113 (8)	0.0171 (8)	0.0164 (8)	−0.0012 (7)	0.0040 (7)	0.0016 (7)
C3	0.0143 (8)	0.0117 (8)	0.0136 (8)	−0.0005 (6)	0.0021 (6)	−0.0008 (6)
C4	0.0125 (8)	0.0132 (8)	0.0159 (8)	−0.0010 (6)	0.0018 (7)	−0.0012 (7)
C5	0.0113 (8)	0.0179 (9)	0.0169 (8)	−0.0005 (7)	0.0027 (7)	0.0021 (7)
C6	0.0153 (9)	0.0137 (8)	0.0138 (8)	−0.0017 (7)	0.0023 (7)	−0.0015 (6)
C7	0.0256 (10)	0.0226 (10)	0.0184 (9)	0.0004 (8)	0.0041 (8)	0.0001 (8)
C8	0.0305 (11)	0.0302 (11)	0.0192 (10)	0.0039 (9)	0.0062 (8)	−0.0048 (8)
C9	0.0334 (12)	0.0244 (10)	0.0243 (10)	0.0072 (9)	0.0025 (9)	−0.0086 (8)
C10	0.0270 (11)	0.0169 (9)	0.0247 (10)	0.0042 (8)	−0.0007 (8)	−0.0036 (8)
C11	0.0151 (9)	0.0160 (9)	0.0176 (9)	0.0016 (7)	−0.0020 (7)	−0.0016 (7)
C12	0.0153 (9)	0.0152 (9)	0.0185 (9)	−0.0004 (7)	−0.0016 (7)	0.0006 (7)
C13	0.0263 (11)	0.0157 (9)	0.0257 (10)	−0.0017 (8)	0.0010 (8)	0.0003 (8)
C14	0.0334 (12)	0.0221 (10)	0.0278 (11)	−0.0061 (9)	0.0030 (9)	0.0045 (8)
C15	0.0319 (12)	0.0280 (11)	0.0226 (10)	−0.0043 (9)	0.0076 (9)	0.0033 (8)
C16	0.0259 (10)	0.0218 (10)	0.0194 (9)	−0.0003 (8)	0.0042 (8)	−0.0004 (8)
C17	0.0538 (17)	0.0398 (14)	0.0322 (13)	−0.0009 (12)	0.0117 (12)	−0.0033 (11)
C18	0.116 (3)	0.0443 (17)	0.0475 (18)	0.0256 (19)	0.038 (2)	0.0151 (14)
C19	0.067 (2)	0.0472 (17)	0.059 (2)	−0.0157 (16)	0.0020 (16)	0.0035 (15)
C20	0.133 (4)	0.0471 (19)	0.051 (2)	−0.001 (2)	0.020 (2)	−0.0120 (16)

### Geometric parameters (Å, º)

**Table:**

Mn1—O1	2.1796 (14)	C11—C12	1.488 (3)
Mn1—O2	2.1546 (14)	C12—C13	1.391 (3)
Mn1—O3^i^	2.1511 (14)	C13—C14	1.387 (3)
Mn1—O4^i^	2.1782 (14)	C14—C15	1.380 (3)
Mn1—N1	2.2473 (16)	C15—C16	1.384 (3)
Mn1—N2	2.2398 (16)	C17—C18	1.488 (4)
Cl1—C2	1.7304 (18)	C19—C20	1.471 (5)
Cl2—C5	1.7322 (18)	O5—H1	0.840
O1—C1	1.254 (2)	O5—H4	0.840
O2—C6	1.258 (2)	O6—H2	0.840
O3—C3	1.257 (2)	O6—H3	0.840
O3—Mn1^ii^	2.1510 (13)	C7—H7	0.950
O4—C4	1.257 (2)	C8—H8	0.950
O4—Mn1^ii^	2.1782 (14)	C9—H9	0.950
O5—C17	1.437 (3)	C10—H10	0.950
O6—C19	1.402 (4)	C13—H13	0.950
N1—C7	1.346 (3)	C14—H14	0.950
N1—C11	1.350 (2)	C15—H15	0.950
N2—C16	1.340 (3)	C16—H16	0.950
N2—C12	1.351 (2)	C17—H17A	0.990
C1—C2	1.400 (3)	C17—H17B	0.990
C1—C6	1.544 (2)	C18—H18A	0.980
C2—C3	1.392 (3)	C18—H18B	0.980
C3—C4	1.541 (2)	C18—H18C	0.980
C4—C5	1.395 (3)	C19—H19A	0.990
C5—C6	1.392 (3)	C19—H19B	0.990
C7—C8	1.388 (3)	C20—H20A	0.980
C8—C9	1.380 (3)	C20—H20B	0.980
C9—C10	1.391 (3)	C20—H20C	0.980
C10—C11	1.392 (3)	H2—H2^iii^	1.0155 (1)
			
O3^i^—Mn1—O2	151.46 (5)	N2—C12—C11	116.06 (16)
O3^i^—Mn1—O4^i^	74.60 (5)	C13—C12—C11	122.40 (17)
O2—Mn1—O4^i^	88.83 (5)	C14—C13—C12	118.93 (19)
O3^i^—Mn1—O1	89.74 (5)	C15—C14—C13	119.5 (2)
O2—Mn1—O1	74.62 (5)	C14—C15—C16	118.5 (2)
O4^i^—Mn1—O1	111.37 (6)	N2—C16—C15	122.82 (19)
O3^i^—Mn1—N2	105.80 (6)	O5—C17—C18	112.5 (2)
O2—Mn1—N2	96.82 (5)	O6—C19—C20	113.3 (3)
O4^i^—Mn1—N2	89.11 (6)	C17—O5—H1	109.5
O1—Mn1—N2	157.24 (6)	C17—O5—H4	109.5
O3^i^—Mn1—N1	98.26 (5)	C19—O6—H2	109.5
O2—Mn1—N1	104.92 (6)	C19—O6—H3	109.5
O4^i^—Mn1—N1	158.45 (6)	N1—C7—H7	118.629
O1—Mn1—N1	88.57 (6)	C8—C7—H7	118.631
N2—Mn1—N1	73.05 (6)	C7—C8—H8	120.730
C1—O1—Mn1	116.52 (12)	C9—C8—H8	120.716
C6—O2—Mn1	117.46 (12)	C8—C9—H9	120.253
C3—O3—Mn1^ii^	117.58 (12)	C10—C9—H9	120.256
C4—O4—Mn1^ii^	116.71 (12)	C9—C10—H10	120.608
C7—N1—C11	118.46 (17)	C11—C10—H10	120.605
C7—N1—Mn1	124.09 (13)	C12—C13—H13	120.536
C11—N1—Mn1	117.40 (12)	C14—C13—H13	120.531
C16—N2—C12	118.74 (17)	C13—C14—H14	120.253
C16—N2—Mn1	123.70 (13)	C15—C14—H14	120.257
C12—N2—Mn1	117.55 (12)	C14—C15—H15	120.770
O1—C1—C2	125.27 (17)	C16—C15—H15	120.765
O1—C1—C6	115.62 (16)	N2—C16—H16	118.596
C2—C1—C6	119.11 (16)	C15—C16—H16	118.588
C3—C2—C1	121.31 (16)	O5—C17—H17A	109.084
C3—C2—Cl1	119.45 (14)	O5—C17—H17B	109.087
C1—C2—Cl1	119.14 (14)	C18—C17—H17A	109.089
O3—C3—C2	125.04 (17)	C18—C17—H17B	109.086
O3—C3—C4	115.60 (16)	H17A—C17—H17B	107.842
C2—C3—C4	119.36 (16)	C17—C18—H18A	109.466
O4—C4—C5	125.24 (17)	C17—C18—H18B	109.469
O4—C4—C3	115.39 (16)	C17—C18—H18C	109.469
C5—C4—C3	119.37 (16)	H18A—C18—H18B	109.486
C6—C5—C4	121.33 (17)	H18A—C18—H18C	109.468
C6—C5—Cl2	119.44 (14)	H18B—C18—H18C	109.470
C4—C5—Cl2	119.23 (14)	O6—C19—H19A	108.909
O2—C6—C5	125.20 (17)	O6—C19—H19B	108.914
O2—C6—C1	115.45 (16)	C20—C19—H19A	108.916
C5—C6—C1	119.36 (16)	C20—C19—H19B	108.918
N1—C7—C8	122.74 (19)	H19A—C19—H19B	107.740
C9—C8—C7	118.6 (2)	C19—C20—H20A	109.468
C8—C9—C10	119.49 (19)	C19—C20—H20B	109.476
C9—C10—C11	118.79 (19)	C19—C20—H20C	109.478
N1—C11—C10	121.97 (18)	H20A—C20—H20B	109.465
N1—C11—C12	115.88 (16)	H20A—C20—H20C	109.470
C10—C11—C12	122.15 (18)	H20B—C20—H20C	109.470
N2—C12—C13	121.54 (18)	O6^iii^—H2—H2	161.01 (15)

Symmetry codes: (i) *x*+1/2, −*y*+1/2, *z*+1/2; (ii) *x*−1/2, −*y*+1/2, *z*−1/2; (iii) −*x*, −*y*+1, −*z*+1.

### Hydrogen-bond geometry (Å, º)

**Table:**

*D*—H···*A*	*D*—H	H···*A*	*D*···*A*	*D*—H···*A*
O5—H1···O6	0.84	1.91	2.716 (4)	160
O5—H4···O5^iv^	0.84	2.00	2.715 (4)	142
O6—H2···O6^iii^	0.84	1.83	2.661 (3)	170
O6—H3···O5	0.84	1.90	2.716 (4)	162

Symmetry codes: (iii) −*x*, −*y*+1, −*z*+1; (iv) −*x*+1, −*y*+1, −*z*+1.

## References

[bb1] Burla, M. C., Caliandro, R., Camalli, M., Carrozzini, B., Cascarano, G. L., De Caro, L., Giacovazzo, C., Polidori, G., Siliqi, D. & Spagna, R. (2007). *J. Appl. Cryst.* **40**, 609–613.

[bb2] Decurtins, S., Schmalle, H. W., Zheng, L.-M. & Ensling, J. (1996). *Inorg. Chim. Acta*, **244**, 165–170.

[bb3] Deguenon, D., Bernardinelli, G., Tuchagues, J.-P. & Castan, J.-P. (1990). *Inorg. Chem.* **29**, 3031–3037.

[bb4] Kabir, M. K., Kawahara, M., Kumagai, H., Adachi, K., Kawata, S., Ishii, T. & Kitagawa, S. (2001). *Polyhedron*, **20**, 1417–1422.

[bb5] Nagayoshi, K., Kabir, M. K., Tobita, H., Honda, K., Kawahara, M., Katada, M., Adachi, K., Nishikawa, H., Ikemoto, I., Kumagai, H., Hosokoshi, Y., Inoue, K., Kitagawa, S. & Kawata, S. (2003). *J. Am. Chem. Soc.* **125**, 221–232. 10.1021/ja027895v 12515525

[bb6] Rigaku (1998). *REQAB* Rigaku Corporation, Tokyo, Japan.

[bb7] Rigaku (2008). *CrystalClear* Rigaku Corporation, Tokyo, Japan.

[bb8] Rigaku (2010). *Crystal Structure* Rigaku Corporation, Tokyo, Japan.

[bb9] Sheldrick, G. M. (2008). *Acta Cryst.* A**64**, 112–122. 10.1107/S0108767307043930 18156677

[bb10] Zheng, L.-M., Schmalle, H. W., Huber, R. & Decurtins, S. (1996). *Polyhedron*, **15**, 4399–4405.

